# Words matter: a qualitative investigation of which weight status terms are acceptable and motivate weight loss when used by health professionals

**DOI:** 10.1186/1471-2458-11-513

**Published:** 2011-06-29

**Authors:** Cindy M Gray, Kate Hunt, Karen Lorimer, Annie S Anderson, Michaela Benzeval, Sally Wyke

**Affiliations:** 1 Alliance for Self Care Research, School of Nursing, Midwifery and Health, University of Stirling, Stirling, FK9 4LA, UK; 2 MRC/CSO Social and Public Health Sciences Unit, 4 Lilybank Gardens, Glasgow, G12 8RZ, UK; 3 Institute for Applied Health Research, Glasgow Caledonian University, Cowcaddens Road, Glasgow, G4 0BA, UK; 4 Centre for Public Health Nutrition Research, University of Dundee, Dundee, DD1 4HN, UK; 5 Institute for Health and Wellbeing, College of Social Sciences, University of Glasgow, G12 8QQ, UK

## Abstract

**Background:**

Health professionals have an important role to play in the management of obesity, but may be unsure how to raise weight issues with patients. The societal stigma associated with excess weight means that weight status terms may be misunderstood, cause offence and risk upsetting patient-professional relationships. This study investigated the views of people who were overweight or obese on the acceptability of weight status terms and their potential to motivate weight loss when used by health professionals.

**Methods:**

A qualitative study comprising 34 semi-structured interviews with men and women in their mid-to-late 30s and 50s who were overweight or obese and had recently been informed of their weight status. Thematic framework analysis was conducted to allow the systematic comparison of views by age, gender and apparent motivation to lose weight.

**Results:**

Although many people favoured 'Overweight' to describe their weight status, there were doubts about its effectiveness to motivate weight loss. Terms including 'BMI' ('Body Mass Index') or referring to the unhealthy nature of their weight were generally considered acceptable and motivational, although a number of men questioned the validity of BMI as an indicator of excess weight. Participants, particularly women, felt that health professionals should avoid using 'Fat'. Whilst response to 'Obese' was largely negative, people recognised that it could be appropriate in a health consultation. Some younger people, particularly those who appeared motivated to lose weight, felt 'Obese' could encourage weight loss, but it was also clear the term could provoke negative emotions if used insensitively.

**Conclusions:**

Although most people who are overweight or obese accept that it is appropriate for health professionals to discuss weight issues with patients, there is great variation in response to the terms commonly used to describe excess weight. There is no one-size-fits-all approach to discussing weight status: some men and younger people may appreciate a direct approach, whilst others need to be treated more sensitively. It is therefore important that health professionals use their knowledge and understanding to select the terms that are most likely to be acceptable, but at the same time have most potential to motivate each individual patient.

## Background

The proportion of people who are overweight or obese has reached unprecedented levels in the UK. In 2008 around a quarter of adults were obese (24.5% in England; 25.7% in Scotland) and a further third were overweight (37.0% in England; 37.3% in Scotland) [1,2]. Being overweight or obese increases risk of ill health. People who are obese are 20 times more likely to develop type 2 diabetes compared to people of normal weight, and 85% of people with hypertension have a BMI (Body Mass Index) over 25 kg/m^2 ^[3]. Excess weight is a risk factor for coronary heart disease, stroke, dyslipidaemia, osteoarthritis and a number of cancers, including cancers of the breast, colon, rectum, endometrium, gallbladder, kidney, oesophagus and pancreas [3]. Current evidence suggests obesity is the second most important preventable cause of cancer after smoking [4]. In 2007 the cost of overweight and obesity to society in terms of illness, sick leave, unemployment and premature death was estimated at £15.8 billion a year and forecast to rise to £49.9 billion by 2050 [3]. This economic burden, together with the considerable health benefits of maintaining a healthy weight, means that management of overweight and obesity is a high priority in the UK.

With the average British adult now categorised as overweight [1,2], people are becoming less able to distinguish between normal and excess weight, and may be in denial about their own weight [5]. A weight loss consultation with a health professional can increase people's understanding and motivate them to lose weight [6-8]. The National Institute for Health and Clinical Excellence and the Scottish Intercollegiate Guidelines Network have recently published comprehensive clinical guidelines for the assessment, treatment and support of patients who are overweight and obese [9,10]. However, excess weight has negative connotations for many people [11-14], even if they are overweight or obese themselves [15]. Western societies have long devalued and stigmatised people who are viewed as 'Fat' [16-19]. Obesity is often associated with some character flaw (e.g. laziness) or moral failing on the part of the individual [20,21]. Adults who are overweight or obese are more likely to report instances of discrimination than people of normal weight [22-24]; for example, longitudinal research has demonstrated that overweight girls who retain excess body fat into early adulthood are less likely to be in employment and have a current partner [25].

Health professionals are aware of the potential for stigmatisation [18,19], and many appear reluctant to broach the subject of weight status even with people who are obese [6,24,26,27]. Challenges include lack of training in the skills required for sensitive weight management counselling [7,28,29] and lack of understanding of the impact, both positive and negative, of using different terms to inform people of their personal weight status [30]. It is clear that some terms may be misunderstood, causing patients upset or offence, and may have potential to jeopardise patient-professional relationships, as well as making patients less likely to engage with offers of advice and support to lose weight. In 2008 the former Chief Medical Officer for England warned that the term 'Obesity' is the new 'Cancer' - "a word that is taboo, that intimidates, that strikes fear" [31]. In an online survey of overweight people (predominantly women) in the UK, the terms 'Fat' and 'Obese' were perceived as offensive and insulting [32]. Likewise, men and women seeking treatment for weight loss in the USA generally viewed the terms 'Fatness', 'Excess Fat' and 'Obesity' as undesirable, with some people (particularly women) also objecting to the terms 'Large Size' and 'Heaviness'[33,34]. Meanwhile, obese adults in Australia found the term 'Fat' more acceptable than 'Obese' [24].

Whilst health professionals may avoid using weight status terms that could be seen as derogatory [34], evidence is emerging that some people may be motivated to lose weight by the terms they find less acceptable. Men attending a weight management group in central Scotland said being told they were 'Obese' by a health professional was one of their main reasons for wanting to lose weight [35]. Government and general practitioner (GP) representatives in the UK have recently suggested telling people they are 'Fat' will encourage them to face up to their problem and take personal responsibility for losing weight [36].

It is clear, therefore, that when thinking about how best to describe a person's weight status and discuss their options for weight management, health care professionals should go beyond simple consideration of which terms are least offensive. Clinicians need to have a fuller understanding of how response to terms may vary (e.g. between men and women or between different age groups). Previous research has focussed on the views of people who were motivated to lose weight [24,32-34], but it is equally important to consider the views of those who do not appear to be motivated as health professionals may experience most difficulty in broaching weight status with these patients. This paper reports data from a qualitative study that aimed to investigate the views of people who were overweight or obese on the acceptability of different weight status terms and their potential to motivate weight loss when used by a health professional.

## Methods

Semi-structured face-to-face and telephone interviews were conducted with a subsample of participants from the 'West of Scotland Twenty-07 Study: Health in the Community', a 20-year longitudinal investigation of social inequalities in health [37]. Interviews were conducted between January and July 2009: ethical permission was obtained from the University of Glasgow Faculty of Law, Business and Social Sciences Ethics Committee.

The Twenty-07 Study has followed three age cohorts born 20 years apart (early 1970s, 1950s and 1930s). Detail on the study can be found elsewhere [37], but briefly the five waves of data collection included a face-to-face interview and a range of physical measures by a trained nurse interviewer. Participants were offered a feedback letter, which included their personal measurements (height, weight, BMI, body fat percentage), following the fifth wave of data collection in 2007/08. The letter also provided some context to allow recipients to interpret their results. For example, people with BMI ≥ 27 kg/m^2 ^were told *"This suggests that you might be overweight"*. This BMI cut-off, which is higher than the standard overweight BMI cut-off (≥ 25 kg/m^2^) [38], was recommended by Twenty-07 clinical advisors. Further detail on the feedback letter is reported elsewhere [39].

Twenty-07 participants were eligible to take part in the current study ('Weight Status Terms Study') if they had taken part in the 2007/08 wave of data collection, had given permission to be re-contacted for further research, had received a feedback letter in the previous six months and were members of the 1950s and 1970s cohorts (aged mid-to-late 50s and mid-to-late 30s at the time of the interviews). The 1930s cohort (aged mid-to-late 70s at the time of the interviews) was excluded as the main focus of the Weight Status Terms Study was the primary prevention of long term conditions associated with excess body weight, and at this age the complexity of associated health problems means a different approach to prevention, which goes beyond the scope of this study, would often be required. The exclusion criteria for the Weight Status Terms Study were that participants had been excluded from one or more Twenty-07 Study measurements according to the fieldwork protocol (e.g. women who were pregnant), had received their feedback letter more than 3 months after their Twenty-07 interview, or had received a 'special' feedback letter highlighting a result (e.g. vitamin deficiency) that the Twenty-07 Study's clinical advisors felt was important to communicate.

Written invitations to take part in the Weight Status Terms Study were sent to a random sample of eligible Twenty-07 participants. We aimed to recruit 32 people, with equal numbers of: men and women; people in their mid-to-late 30s and people in their mid-to-late 50s; and people whose BMI was in overweight and obese ranges. People with a normal BMI were also recruited, but as the focus of the Weight Status Terms Study was on excess weight, their views are not presented here. We also sought to conduct equal numbers of face-to-face and telephone interviews (both n = 16) to explore any potential respondent bias that might have been associated with the visible weight status of the two researchers (both normal BMI). Informed consent was obtained in writing from face-to-face interview participants and audio-recorded for telephone interview participants. People not selected for interview were sent a letter to thank them for their interest in the study.

All but three interviews were carried out with the participant at home (two face-to-face interviews were conducted in university settings; one telephone interview was conducted while the participant was a passenger in a car). The interviews lasted 33-90 minutes, during which participants were either handed a list of weight status terms (face-to-face interviews) or wrote down each term as the interviewer read it out (telephone interviews). The list of weight status terms (shown in Table 1) was previously used in an online survey of overweight and obese people in the UK [32]. The interviews followed a semi-structured format that explored people's views on the *acceptability *of the weight status terms, both generally and specifically when used by a health professional, and on their *effectiveness *in motivating lifestyle changes.

**Table 1:** List of weight status terms used in interviews

Weight status terms
'Overweight'

'Heavy'

'Obese'

'High BMI'

'Excessive Weight'

'Fat'

'Excessive Fat'

'Large'

'Unhealthily High Body Weight'

'Weight Problem'

'Unhealthy BMI'

Participants were also asked about their views of the feedback letter from the Twenty-07 Study, their knowledge of the health risks associated with excess weight, and their suggestions for addressing the increasing prevalence of overweight and obesity in society. Some of these data (including the views of people with normal BMI) are presented elsewhere [39]. Interviews were audio-recorded with participants' consent and transcribed verbatim.

Transcripts were analysed using the Framework Approach [40], where data are coded, indexed, charted systematically, then organised using matrices in which each participant is represented by a row and each theme by a column. NVivo8 software was used to assist data coding and organisation. The coding frame was based on our main research questions, but also allowed for any unanticipated themes to be systematically identified and explored. Summary analyses of three key themes are relevant here. *Response to terms*, which included the acceptability/unacceptability of the terms to participants in general; most data for this theme were elicited in response to the question "How would you feel if someone used that term about you?". *Terms and health professionals*, which included participants' actual or anticipated response to terms if used by a doctor or a nurse; most data for this theme were elicited in response to the question "How would you feel if that term were used by a doctor or nurse?". *Terms and effectiveness*, which included the motivational value of the terms if used by a health professional in the context of a consultation; most data for this theme were elicited in response to the question "Which of these terms do you think doctors or nurses should use if they want to encourage people to lose weight?". Four transcripts were cross-coded to ensure that the coding framework had been applied consistently.

Data were summarised for each theme using an adapted One Sheet Of Paper analysis [41] to ensure that all issues and views were represented, and to allow differences in response according to age, gender and BMI to be explored. (Initial readings of the transcripts did not reveal any difference in response between face-to-face and telephone interviews, therefore no further analysis of response by interview type was undertaken). As analysis progressed, no differences between people who were overweight and those who were obese were detected; however, possible differences between those who appeared to be motivated to lose weight and those who appeared unmotivated did emerge. We therefore attempted to group participants by their apparent motivational status by rereading the transcripts and indexing every mention of existing weight loss or firm intention to lose weight with concrete plans (e.g. attending a weight management group or visiting a health professional for advice). Participants were then assigned to one of three 'motivational status' groups: 'Appears Motivated', 'Appears Unmotivated' or 'Unclear'. Assignation was conducted independently by two researchers: agreement was 100% after discussion. The final stage of analysis investigated differences or similarities in accounts by age, gender and apparent motivation to lose weight.

This paper presents summary analyses. The extracts used are labelled to indicate the participant's ID ('I' = face-to-face interview or 'T' = telephone interview), age ('35+' = mid-to-late 30s or '55+' = mid-to-late 50s), gender ('man' or 'woman'), weight status ('overweight' or 'obese') and motivational status ('motivated' = Appears Motivated, 'unmotivated' = Appears Unmotivated).

## Results

### Participant characteristics

Of the 263 overweight or obese Twenty-07 participants invited, 48 (18.3%) replied and 34 were interviewed. (The body fat composition of the four overweight men in their mid-to-late 30s who were orginally interviewed was normal, therefore to ensure a broad range of views was represented, two additional interviews were conducted with men in this group whose body fat was high.) Most of the 34 participants interviewed (76.5%) had taken part in all five waves of data collection in the Twenty-07 Study and many (64.7%) were from professional and managerial households. Overall response was lower than expected, particularly amongst the younger men and women who were obese (16.2%). Table 2 shows the number of interviews conducted by age, BMI, gender and apparent motivation. Of those who responded but were not interviewed: one person did not remember receiving the Twenty-07 feedback letter and was excluded; three could not be contacted; and 10 were not invited to be interviewed as recruitment targets had been reached.

**Table 2:** Participant profile

Group	Appears motivated	Appears unmotivated	Motivation unclear	Total
Overweight, men, aged 35+	2	4	0	6

Overweight, men, aged 55+	2	1	1	4

Overweight, women, aged 35+	3	1	0	4

Overweight, women, aged 55+	2	2	0	4

Obese, men, aged 35+	3	0	1	4

Obese, men, aged 55+	0	3	1	4

Obese, women, aged 35+	3	1	0	4

Obese, women, aged 55+	3	1	0	4

**Total**	**18**	**13**	**3**	**34**

### General response to terms

Participants did not discuss every term on the list, just those that seemed most salient to them. Most people mentioned 'Fat' unprompted; most also discussed 'Obese' and 'Overweight', sometimes in response to prompts from the interviewer. Many people thought that 'Overweight' or 'Heavy' would be the most acceptable way for someone to describe their current weight status and they often used these terms to describe themselves:

I know I'm overweight because I'm not my ideal dress size; I'm not my ideal weight that I want to be, so I would term myself as being overweight. And a lot of women in my situation are maybe a dress size higher than they want to be, you know, they're a fourteen instead of a twelve; they class themselves as overweight (T03, 35+ woman, obese, unmotivated).

'Large', 'High BMI', 'Unhealthy BMI' and 'Excessive Weight' were also often endorsed as acceptable terms for general use. However, some men, particularly those who appeared unmotivated to lose weight, argued that BMI was an inaccurate way to measure weight status:

I've not looked at the science of it, the actual calculation behind it, but I do think if you've got people who have above normal muscle content then they could produce a high BMI. So I am not convinced about that (T05, 35+ man, overweight, unmotivated).

Reaction to the terms 'Obese',' Fat' and 'Excessive Fat' was usually adverse:

Just 'Obese'... just sounds massive, doesn't it? I don't know, it just makes me think of somebody that's really, really big, but then obviously it's not [laughs]. Aye, I think 'Obese' would be the most hurtful (T21, 35+ woman, obese, motivated).

I think 'Fat's' the big one for me. I think that's quite a hurtful comment. [...] Just really, if you were to say to somebody 'You're fat', I think there's a wrong way to take that. It's a horrible thing to say to somebody (T26, 35+ woman, overweight, motivated).

People made moral judgements in relation to 'Fat' and 'Obese'. Both terms were associated with laziness, greed or pity:

'Obese' to me is somebody who's grossly overweight, as you see the unfortunate souls who are on the television who seem to be brought out every so often (I19, 55+ man, overweight, motivated).

Nevertheless, a number of participants, most of whom were young and appeared motivated, used 'Obese' in relation to their own weight, often saying they were categorised as 'Obese' (or close to 'Obese'). 'Fat' was frequently used in social situations: for women the term was either considered neutral or insulting, whereas men often treated it as banter:

There's a group of us, the bowling club, and we all have a standing joke about being bald and being fat, so... and we all slag each other when we put on a few pounds (I02, 35+ man, overweight, unmotivated).

### Response to terms if used by health professionals

Most participants suggested that they would respond differently to weight-related terms used socially compared to during encounters with health professionals. Participants who appeared motivated to lose weight often seemed to engage more in discussion of which terms they would find acceptable from a clinician than those who appeared unmotivated (the one exception to this pattern was 'Fat'). Some, particularly those from the younger age group, were explicit in discussing the difference between terms being used by a health professional as opposed to by a family member or friend:

If it's a doctor or a nurse then they're qualified to say that, whereas if it's a friend I think it's more of an opinion. I would think that's more of an opinion they've got than any kind of medical justification for saying it, whereas if it's a doctor or a nurse then I would certainly listen to them more (T05, 35+ man, overweight, unmotivated).

Many appeared to respect and trust health professionals' knowledge, expertise and authority, and agreed that they should raise weight issues with patients. However, some cautioned sensitivity when doing so:

I think if somebody's in, you know, it's going to take two seconds to say, 'Did you realise that you're a bit overweight?' or, 'You're a bit this or that'. But again, if somebody's in with some heartbreaking news, you can't just say, 'By the way, we've noticed you're a bit fat as well!' (I12, 35+ woman, overweight, motivated).

There was recognition, particularly among younger participants, that 'Obese' was a clinical or a medical term and that the clinical definition of obesity did not necessarily equate with popular perceptions:

I'm educated enough now to know that being clinically obese actually is a lot less than what you would [think] (T05, 35+ man, overweight, unmotivated).

Similarly, the majority of participants in their mid-to-late 30s sanctioned the use of 'Obese' by health professionals. However, opinion was more divided among older people: whilst some agreed that 'Obese' would be acceptable from a health professional, others remained unconvinced:

Calling [patients] 'Obese' I don't think helps. It's not quite insulting but it's getting that way, and it's got nothing to do with health (T09, 55+ man, obese, unmotivated).

Terms that were considered to be generally acceptable, namely 'Overweight', 'High BMI', 'Unhealthy BMI' and 'Unhealthily High Body Weight', were also endorsed for use by clinicians. In contrast, 'Fat' was viewed by some people, particularly women, as being too personal or too judgemental:

I think there's better ways of saying things. [...] I think 'Fat' and 'Excessive Fat' sound critical, whereas the others sound more like constructive criticism (I03, 55+ woman, overweight, motivated).

### Which terms might be motivational to lose weight?

Once again participants who appeared motivated to lose weight were more enthusiastic about expressing their views on this topic than those who appeared unmotivated. 'Unhealthy BMI', 'High BMI' and 'Unhealthily High Body Weight' were often felt to be good terms to motivate weight loss: they were seen as professional and providing a clear definition of the problem. However, some younger participants cautioned that the impact of BMI would depend on people's understanding of the association between body mass index and weight.

The term 'Overweight' was not generally seen to be effective in motivating weight loss, probably because many people (including women) felt comfortable about being described as 'Overweight':

Although I see myself being overweight, I don't kind of stand in front of the mirror and go, 'Oh I hate...' Maybe if I did I would be a bit better at losing weight, but I don't (I12, 35+ woman, overweight, motivated).

In contrast, although 'Obese' was often considered an unacceptable term to use, many people who were in the younger age group and/or appeared motivated thought the term could be effective for encouraging weight loss. Some participants (mostly women) drew on personal experience to illustrate this point:

If I'm with my nurse then she'll talk about weight issue or being a wee bit overweight. I'm not a wee bit overweight, I'm morbidly obese, but no professional that I sat with... my GP at no point told me I was morbidly obese. [...] I think the new one, the new guy, he's really young, I think he mentioned the word 'Obese'; and this was just before I kind of started to lose weight (I06, 35+ woman, obese, motivated).

In the [Twenty-07 feedback] letter it just gave you a number and it said that I was in the overweight category, which I knew I'd put on a wee bit of weight after I'd had my little boy. But then it didn't tell you actually what the ranges were, so it didn't tell you where you were on the range. So I looked it up on the internet and discovered that I was fractions off being classed as obese, which absolutely shocked me. So I have now lost over a stone (I11, 35+ woman, overweight, motivated).

Importantly, there was clear evidence that using 'Obese' inappropriately could be counterproductive. A number of women in particular talked about emotional responses:

I mean if I was obese, right, and I went to a doctor and he said that to me, I would be look, I'm saying to you I would come out and I'd start crying, right. So I would probably go and eat, you see, do you know what I mean? I'd go into depression for a day and then I'd probably say 'Okay, sod him I'm having something; I'm having my sweeties, my crisps or whatever it is' (T02, 55+ woman, overweight, motivated).

I think that was one of the doctors in the [hospital]. [...] They didn't mean it unkindly. I think I had said something like, 'Oh, I don't even want to know what weight I am.' And she said, 'Well, you're actually in the obese category now' she said, 'not morbidly obese, but obese.' I just thought, 'Oh no!', I just wanted the ground to open up and swallow me (I09, 55+ woman, obese, motivated).

'Fat' and 'Large' were not generally considered motivational even when used by a health professional. However, some men claimed terms that could be viewed as hurtful, aggressive or derogatory might encourage them to try to lose weight:

The words that to me, the words that would upset you would probably be the ones that are more likely to get you to do something about it (I02, 35+ man, overweight, unmotivated).

Relating weight status to health emerged as a popular suggestion for motivating weight loss:

I think what would be really useful is if it was made clearer to people who are what they would presume to be overweight, there's actually more of an issue than just being overweight. [...] It has to be put across to people that that's not okay, because you just do tend to think, 'Oh I'm only a dress size more than I should be and that's okay, I can still eat my chocolate, and I can still do what I do and carry on as I am' (T03, 35+ woman, obese, unmotivated).

A number of men also felt that it was important to allow time for a full discussion of the problem, including the health implications and possible solutions:

First of all, get the patient, or whoever it is, to accept that the weight they are is not, the physical condition they're in, is not one that they should stay in [...] and to do that you've got to basically explain to them why, and on what grounds, you decide that their weight is not what it should be. They've got to accept that. You've also got to give them a feasible way of going forward and what they can do about it, and what help they can receive in doing something about it (T16, 55+ man, overweight, motivated).

## Discussion

This study clearly demonstrates the sensitivity of weight status terms for people who are overweight and obese, and indicates that health professionals cannot rely on a single 'one-size-fits-all' approach to discussing excess weight with patients. It is important that clinicians exercise sensitivity in selecting the terms that are most likely to be acceptable, but at the same time have most potential to motivate each individual patient. We think that the lower than expected response to the invitation to take part in the study, particularly from those who were obese, may indicate that people become more reluctant to discuss weight-related issues as their own weight increases. Nevertheless, most participants felt it would be appropriate for health professionals to raise concerns about their weight, and in line with previous findings [6-8], the younger age group in particular said they would be more likely to try to lose weight following discussions with a doctor or nurse than after talking to friends or family members.

There was clear demarcation between response to weight-related terms used in a medical or social context. For example, although 'Obese' was viewed as socially unacceptable, there was recognition, especially among younger people, that it would be appropriate from a health professional. While previous research demonstrated that being described as 'Obese' by a health professional encouraged men to lose weight [35], our study demonstrates that 'Obese' can also be motivational for women. However, a number of people seemed more comfortable with 'Clinically Obese' than 'Obese', suggesting that framing excess weight as a medical problem that can be addressed can also facilitate discussion of appropriate solutions and support [42].

In contrast, 'Fat' was viewed as a social, rather than a medical, term. It was a highly emotive term and most people had a clear opinion about it. Despite recent debate [36], our participants thought that it would be inappropriate for health professionals to use 'Fat' when referring to a patient's weight status and that the term would be unlikely to motivate weight loss. Men were less negative about 'Fat' than women and gave examples of using the term humorously. However, whilst some men might welcome the use of humour to facilitate discussions with health professionals [43], it can act as a barrier to communication by creating ambiguity and confusion about the message being delivered [44,45], and so should be used with caution.

Terms that people found acceptable, such as 'Overweight', were often seen as lacking potential to motivate weight loss. Some men felt they would take more notice of terms that appeared to be hurtful, upsetting and aggressive. Evidence suggests that men tend to favour a direct, decisive and result-oriented style of communication [46-48], therefore playing safe by selecting language that is least likely to cause offense may not always be the most successful approach to encouraging men to lose weight. Allowing time to define the problem, talk it through and then offer potential solutions may be more effective.

Highlighting the health risks associated with excess weight emerged as a popular suggestion for motivating weight loss. However, this finding should be interpreted within the context of optimistic bias, where people tend to underestimate the likelihood that they will develop the health problems that are being discussed. Optimistic bias can mean that being made aware of population-level health risks does not affect individual behaviour [49,50]. Emphasising the potential for personal health benefit is likely to be more effective than general health risk information in motivating weight loss attempts [51].

Our qualitative study extends findings from previous surveys [32-34] by providing an understanding of the range of views and perceptions that are evoked by various weight status terms. Another strength is the inclusion of people who were overweight or obese but who did not appear to be motivated to lose weight. Our results revealed a number of differences between people who we categorised as 'motivated' and 'unmotivated'. The younger participants who appeared motivated were more likely to use 'Obese' or 'Approaching Obese' to describe themselves than those who appeared unmotivated. This finding suggests that having an accurate understanding of personal weight status, rather than just a vague self-perception of being 'Overweight', might help to motivate weight loss.

A number of seemingly unmotivated men questioned the validity of BMI as an indicator of unhealthy weight status. All gave examples of their physical prowess and current or previous physical activity regimens, demonstrating the importance of 'fitness' in defining masculine identity and contributing to men's concept of general well-being [52]. Men may view physical activity as protection from, or compensation for, ill health and negative lifestyle choices (e.g. poor eating habits). Exercise may also allow men to distance themselves from the 'healthist' doctrine of increased body size being a personal and social liability that the individual has a responsibility to overcome [53,54], thereby making them resistant to advice to lose weight.

Finally, participants categorised as motivated to lose weight tended to contribute more to discussions about which terms health professionals should use and their potential effectiveness. This could simply reflect increased engagement in weight-related issues by people who are already motivated; however, it could also demonstrate greater discomfort over talking about weight-related issues among those who are not motivated.

### Limitations

Despite efforts to ensure a range of views, there were no participants from ethnic minorities in the Weight Status Terms Study, reflecting the relative lack of ethnic diversity of the population when the Twenty-07 Study was established. People of lower socioeconomic status were also underrepresented, meaning our findings may not fully reflect the views of overweight and obese people from less affluent households. Our participants were highly motivated to contribute to research: most had remained loyal to the Twenty-07 Study over 20 years. Nevertheless, even in such a generally compliant population, this self-selection produced a low response rate amongst people who were obese. Thus our sample may not have included those who were most uncomfortable about discussing excess weight. The assignation of participants to motivational status groups depended on subjective interpretation of the transcripts. However, this judgement was based on detailed analysis of each transcript, and good agreement between independent researchers reduces the likelihood that participants were wrongly assigned.

## Conclusions

Although health professionals in the UK are being advised to discuss weight issues with patients, the debate over how to do this continues [31,36]. Our study suggests that despite great variation in response to terms describing excess weight, most people feel it is an appropriate topic for their health professional to raise. However, there was disparity between the terms people find most *acceptable *and those that are most likely to be *effective in motivating *them to lose weight. It is therefore important to find language that will allow the problem to be accurately defined, but at the same time will keep the patient engaged and avoid causing offence or upset. Health professionals need to develop the understanding and skills that will allow them to make appropriate choices about which terms to use on an individual basis. Future research should focus on interactions between clinicians and their patients. These studies would provide insight into the mechanisms of effective communication of messages about the prevention and management of obesity, and the role of the health professional in motivating and supporting patients to lose weight and maintain that weight loss long term.

## Competing interests

The authors declare that they have no competing interests.

## Authors' contributions

All authors contributed to the design of the study, read and discussed transcripts, validated the coding frame and commented on drafts of the paper. CMG and KL conducted the interviews. CMG performed the analyses and drafted the manuscript. SW cross-coded a selection of transcripts and carried out independent assignation to motivational status category. All authors read and approved the final manuscript.

## Pre-publication history

The pre-publication history for this paper can be accessed here:

http://www.biomedcentral.com/1471-2458/11/513/prepub
